# The Impact of Job Satisfaction on the Quality of Life of Formal Caregivers of the Elderly

**DOI:** 10.3390/healthcare12232432

**Published:** 2024-12-03

**Authors:** Marina Sousa, Helena Martins, Alexandra R. Costa, Anabela Almeida Silva

**Affiliations:** 1 School of Engineering, Polytechnic of Porto, 4200-465 Porto, Portugal; map@isep.ipp.pt; 2 RESILIENCE—Center for Regional Resilience and Sustainability, Polytechnic Institute of Setúbal, 2910-761 Setúbal, Portugal; helena.martins@esce.ips.pt; 3 CEOS.PP, ISCAP, Polytechnic Institute of Porto, 4465-004 Mamede de Infesta, Portugal; 4 Nova School of Business and Economics, 2775-405 Carcavelos, Portugal; 5 CIETI—Center for Innovation in Engineering and Industrial Technology, 4249-015 Porto, Portugal; 6 Faculty of Economics, University of Porto, 4200-464 Porto, Portugal; anabela.asilva94@gmail.com

**Keywords:** formal caregivers, quality of life (QoL), absenteeism, job satisfaction, elderly care

## Abstract

Background/Objectives: Absenteeism refers to the frequent, often unplanned, absence from the workplace. This study examines the interrelations among job satisfaction, quality of life (QoL), and absenteeism among formal caregivers for elderly individuals. With the significant demographic shift toward an aging population, understanding these dynamics is increasingly important. Methods: A sample of 82 caregivers from Portuguese Private Social Solidarity Institutions was used, with QoL assessed through the WHOQOL-Bref and job satisfaction measured via the Minnesota Satisfaction Questionnaire (MSQ). Results: The results reveal that higher levels of job satisfaction are associated with improved QoL and lower rates of absenteeism. Specifically, caregivers with higher satisfaction reported fewer sick leaves and a more favorable perception of their QoL. Conclusions: These findings underscore the need for supportive workplace policies that enhance caregiver satisfaction, ultimately contributing to both caregiver well-being and the quality of care provided.

## 1. Introduction

According to the World Health Organization (WHO), population aging is accelerating globally and is regarded as one of the most significant demographic trends of the 21st century [1,2]. In 2019, the global population of individuals aged 60 years and older reached 1 billion and is projected to increase to 1.4 billion by 2030 and 2.1 billion by 2050 [3]. This unprecedented acceleration is the result of the combined effect of rising life expectancy and declining birth rates [4,5]. According to the Portuguese National Institute of Statistics (INE, Instituto Nacional de Estatística), Portugal is the European Union country with the highest percentage of elderly people (38.2% for every 100 people aged 15 to 64 years) [6].

Aging is frequently associated with a decline in motor, cognitive, and social abilities, which often leads to increased dependency [7]. Institutionalization, whether partial (e.g., day centers) or full (e.g., nursing homes and senior residences), becomes a valuable option for providing comprehensive support and care. This choice may be made by family members who recognize limitations in their physical, financial, or emotional capacity to support elderly relatives or by elderly individuals themselves who seek an environment better suited to their care needs [5,8,9,10].

The caregiving profession, while essential, imposes a substantial physical, psychological, and professional burden on caregivers. Research by Costa et al. [11] indicates that formal caregivers frequently experience disruptions in their personal lives due to the demands of their role, which can result in physical and mental health challenges. This burden may adversely affect both the quality of care provided and caregivers’ job satisfaction and overall quality of life (QoL) as their fatigue and psychological strain increase [11,12,13].

The concept of QoL is multidimensional, encompassing social, physical, mental, emotional, and spiritual aspects. It emerges as a socially constructed perception of individual comfort [14,15]. The WHO defines QoL as an individual’s perception of their position in life in the context of the culture and value systems in which they live and in relation to their goals, expectations, standards, and concerns [1]. Further research has shown that job satisfaction profoundly impacts both personal well-being and organizational outcomes. Higher job satisfaction correlates with improved health and happiness, reduced turnover and absenteeism, better retention, and lower interpersonal conflict in the workplace [15,16,17]. Studying job satisfaction among caregivers is thus critical, as their well-being directly affects the quality of life of those they care for [18].

Existing research consistently demonstrates a meaningful association between job satisfaction and QoL among formal caregivers of the elderly. Factors such as workload, social support, and training significantly influence caregiver satisfaction, with greater satisfaction linked to lower stress levels and enhanced quality of care [19,20,21]. Despite the inherently stressful nature of caregiving, formal caregivers often report high life satisfaction levels, though burden, fatigue, and insomnia can detract from their QoL [22,23].

**Hypothesis** **1 (H1).** *There is a significant positive correlation between job satisfaction and quality of life (QoL) among formal caregivers for the elderly.*

Absenteeism is defined as the absence of an employee from their workplace during scheduled workdays and can be measured by both the frequency and duration of these absences. Absenteeism may be involuntary, resulting from factors beyond the individual’s control, or voluntary, occurring when the individual elects not to attend work without justification [24]. Among formal caregivers, absenteeism is multifactorial and influenced by factors such as excessive work hours, job dissatisfaction, caregiver competence, and the quality of leadership and available resources. Additionally, personal factors such as commute distance and broader organizational conflicts contribute to work strain and absenteeism [25,26]. Although the relationship between job satisfaction and absenteeism is well documented, findings vary, with some studies indicating a negative correlation, suggesting that higher job satisfaction can mitigate absenteeism [27,28]. This is further supported by the role of resilience in promoting job engagement and reducing burnout among caregivers [29].

**Hypothesis** **2a (H2a).** *There is a significant negative correlation between overall job satisfaction and absenteeism among formal caregivers for the elderly.*

Moreover, both intrinsic motivation and the social environment at work play crucial roles in the relationship between job satisfaction and absenteeism. Informal caregivers, for example, experience increased absenteeism due to family–work conflicts and emotional exhaustion [29]. Conversely, extrinsic factors such as pay and working conditions also have a significant impact on job satisfaction and absenteeism [30].

**Hypothesis** **2b (H2b).** *There is a significant negative correlation between intrinsic job satisfaction and absenteeism among formal caregivers for the elderly.*

**Hypothesis** **2c (H2c).** *There is a significant negative correlation between extrinsic job satisfaction and absenteeism among formal caregivers for the elderly.*

The complex relationship among caregiving burdens, absenteeism, training, and social support emphasizes the need for adequate support mechanisms for caregivers. Well-trained caregivers who receive substantial social support can better handle the demands of their work, thereby enhancing their QoL and reducing absenteeism. Conversely, inadequate training exacerbates caregiver burden, leading to higher absenteeism and diminished care quality [19,20].

**Hypothesis** **3 (H3).** *There is a significant negative correlation between quality of life and absenteeism among formal caregivers for the elderly.*

In summary, the literature underscores a complex interrelationship among job satisfaction, QoL, and absenteeism among formal caregivers for the elderly, warranting further investigation.

**Figure 1:**
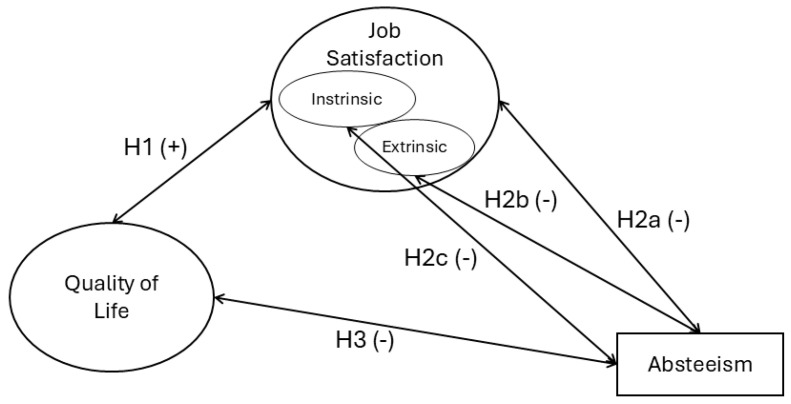
Research model and hypotheses, (+/-) refers to the hypothesized positive/negative relationship between the variables.

This study aims to analyze the associations between QoL and job satisfaction among formal caregivers of elderly individuals. This research used a survey questionnaire to gather empirical evidence, aiming to generate insights and ideas for targeted interventions that reduce the natural strain caregivers experience and enhance their quality of life. The study’s hypotheses are outlined in Figure 1.

## 2. Materials and Methods

In this study, a quantitative research design with convenience sampling was utilized through a survey questionnaire. The objective was to gather empirical evidence to generate insights and propose specific interventions aimed at reducing the occupational strain naturally experienced by caregivers, thereby contributing to an improvement in their quality of life (QoL). Data collection was conducted online via Google Forms, and individual participation requests were distributed by email to employees of Portuguese Private Social Solidarity Institutions (IPSS) who are formal caregivers working in Portugal. Eligible participants were required to have direct caregiving responsibilities for elderly individuals for at least 10 h per week. The invitation email was formatted as a cover letter, briefly explaining the study’s purpose, ensuring response confidentiality.

A self-administered, voluntary, anonymous, and confidential questionnaire was employed for data collection. The sample was selected based on convenience criteria, allowing for easy access, availability, and willingness to participate among eligible respondents.

The questionnaire was divided into three sections. The first section included 14 sociodemographic questions, one of which was optional, covering aspects such as age, gender, educational background, absenteeism, employment duration, type of employment contract, weekly work hours, and monthly income. The second and third sections comprised standardized instruments: the WHOQOL-Bref for assessing the caregivers’ quality of life and the MSQ for evaluating job satisfaction.

Permission to use the WHOQOL-Bref was obtained from the team responsible for its validation within the Portuguese context. Formal authorization for the MSQ was not required, as it is publicly available online.

A total of 90 responses were collected, of which 8 were excluded due to unmet inclusion criteria (e.g., not working a minimum of 10 h per week with elderly individuals or lacking direct caregiving duties). Specifically, one respondent was excluded for not working in Portuguese territory, one for not working in an IPSS, two for lacking direct contact with elderly individuals, and four for working fewer than 10 h per week. This resulted in a final sample of 82 respondents.

The data collected via the online platform were entered into an Excel database and subsequently transferred to SPSS version 28 for analysis. Statistical analyses were conducted using SPSS, with Pearson correlation tests employed to explore the relationships within the data.

### 2.1. WHOQOL-Bref

Assessment of quality of life can be conducted using the World Health Organization Quality of Life (WHOQOL) scale, developed by the WHOQOL Group. This generic, multidimensional, and culturally adaptable measure is structured into six quality of life domains: physical, psychological, level of independence, social relationships, environment, and spirituality/religion/personal beliefs. The original version, known as the WHOQOL-100, consists of 100 questions that include 24 specific facets within each domain, along with a general facet evaluating overall satisfaction with quality of life and general health perception. Responses are collected using a 5-point Likert scale [31].

In this study, the shortened Portuguese version of the scale, the WHOQOL-Bref [32], is used. This version offers a valid and reliable alternative to the WHOQOL-100 [32] and contains 26 questions across four domains: physical, psychological, social relationships, and environment. Each domain is summarized by 24 facets that encapsulate quality of life aspects relevant to that domain, while also providing a global indicator of overall satisfaction with quality of life.

### 2.2. Minnesotta Satisfaction Questionnaire (MSQ)

Job satisfaction is influenced by a wide range of factors, and one of the most widely used instruments for measuring it is the Minnesota Satisfaction Questionnaire (MSQ) [33]. The full MSQ scale consists of 100 questions that assess 20 dimensions of job satisfaction. This questionnaire has high reliability and validity and is internationally recognized for measuring job satisfaction. Responses are collected on a 5-point Likert scale.

In this study, the MSQ-Short Form was used, consisting of 20 questions selected from the original MSQ to best represent each of the 20 dimensions [33]. This version has been shown to be applicable to the Portuguese population [34]. In this formulation, job satisfaction is evaluated across two dimensions: intrinsic satisfaction (related to the job itself, encompassing factors such as responsibility, personal growth, achievement, etc.) and extrinsic satisfaction (associated with factors such as status, pay, and general working conditions).

The short version of the MSQ was used, consisting of 20 questions, divided into two domains: the Intrinsic domain, with ten questions, and the Extrinsic domain, also with ten questions. Each item is answered on a five-point scale: 1—Very dissatisfied with this aspect of my job; 2—Dissatisfied with this aspect of my job; 3—Can’t decide if I’m satisfied or dissatisfied with this aspect of my job; 4—Satisfied with this aspect of my job; 5—Very satisfied with this aspect of my job. Responses are totaled to provide a composite score, where higher scores indicate higher levels of job satisfaction.

## 3. Results

### 3.1. Sample

In addition to the two instruments described, a set of questions was included at the beginning of the questionnaire to enable the socio-demographic characterization of the sample. The results are presented in Table 1.

The analysis of the data in Table 2 indicates that the sample consists primarily of female participants, with ages ranging from 20 to 61 years and an average age of 35. Most participants are married, and the majority hold higher education qualifications.

Regarding roles within the institutions, the largest professional group—auxiliary staff—includes individuals responsible for personal hygiene, feeding, administering medication, assisting with dressing, supporting physical mobility, and facilitating communication with the elderly in their care. This group is followed by professionals with higher education in health-related fields, including nurses, physiotherapists, and gerontologists. The administrative category includes roles such as financial director, clerk, coordinators, and administrative technicians. Lastly, the “other” category encompasses cultural animators, social workers, and psychologists.

Respondents reported a high level of confidence in their training adequacy when asked if they felt sufficiently prepared to provide the necessary care for the elderly (mean = 4.1; standard deviation (SD) = 0.76). 

On average, they have been employed at the institution for 6.17 years and work approximately 37 h per week.

An analysis of the data in Table 3 reveals that most individuals did not take any sick leave days in the past year, with an overall average of 1.24 days.

A question regarding remuneration was also included; however, as it was optional, several respondents chose not to answer. Among those who did respond, salaries ranged from EUR 300 to EUR 1201, with an average of EUR 720.

### 3.2. Minnesota Satisfaction Questionnaire (MSQ)

Cronbach’s Alpha was calculated to assess the reliability of the measurement instrument, yielding a value of α = 0.95. This result, being greater than 0.7, indicates that the scale is reliable for use.

When analyzing the results of the individual questions, it is noteworthy that the highest-scoring item (mean = 4.46) belongs to the intrinsic domain and is “The opportunity to do things for others”. Conversely, the lowest-scoring item (mean = 2.70) falls within the extrinsic domain and is “The relationship between pay and the amount of work I do”. Table 4 provides a description of the job satisfaction variable and its respective domains.

Based on the results in Table 4, overall job satisfaction is above the average value of the scale, with intrinsic satisfaction averaging higher than extrinsic satisfaction.

### 3.3. WHOQOL-Bref

The questionnaire consists of four domains: the physical domain, containing seven questions; the psychological domain, with six questions; the social relations domain, comprising three questions; and the environmental domain, with eight questions. The questions are organized on 5-point Likert-type scales, measuring intensity, frequency, capacity, and evaluation.

Cronbach’s Alpha was calculated to assess the reliability of the measurement instrument, yielding a value of α = 0.924. Since this value is greater than 0.7, it indicates that the scale is reliable for use.

An individual analysis of the questions reveals that the parameters most positively influencing the perception of quality of life include the absence of the need for medical care to carry out daily activities (mean = 4.22), the perception of enjoyment in life (mean = 4.22), and the ability to move around independently (mean = 4.44). Conversely, the parameters that most negatively impact quality of life perception are insufficient financial resources to meet one’s needs (mean = 3.04), dissatisfaction with sleep (mean = 3.07), and limited opportunities for leisure activities (mean = 3.10). Table 5 provides a detailed description of the quality of life variable and its domains.

Based on the results in Table 5, it can be concluded that overall quality of life is above the scale’s midpoint, with the environmental domain showing the lowest average value, while the other domains have similar average values.

Table 6 presents Pearson’s correlations between the two variables under study and their respective domains.

An analysis of Table 6 reveals that the variables under study—job satisfaction, quality of life, and their respective domains—are positively correlated, with statistically significant correlations. This finding suggests that individuals who report higher levels of job satisfaction, including their domains, also tend to report higher levels of quality of life and its domains.

### 3.4. Testing Hypothesis

The analysis confirmed Hypothesis 1, demonstrating a positive correlation between job satisfaction and all domains of quality of life among formal caregivers for the elderly. Specifically, the correlations were as follows: physical domain (r_(80)_ = 0.336, *p* < 0.01; psychological domain r_(80)_ = 0.370, *p* < 0.01; relationship domain r_(80)_ = 0.265, *p* < 0.05; and environmental domain r_(80)_ = 0.515, *p* < 0.01. These results suggest that higher job satisfaction is associated with better caregiver quality of life across multiple domains.

Support was found for Hypotheses 2a and 2b, indicating that job satisfaction negatively correlates with absenteeism. The correlation coefficients were as follows: overall job satisfaction (r_(80)_ = −0.241, *p* < 0.05) and intrinsic job satisfaction (r_(80)_ = −0.273, *p* < 0.05), reflecting a moderate inverse relationship. However, Hypothesis 2c was not supported, as no significant correlation was found between extrinsic job satisfaction and absenteeism, (r_(80)_ = −0.194, *p* = 0.081).

The results also partially supported Hypothesis 3, showing that higher quality of life is associated with lower absenteeism, with significant negative correlations in several domains: physical domain (r_(80)_ = −0.314, *p* < 0.01); psychological domain (r_(80)_ = −0.339, *p* < 0.01); and environmental domain (r_(80)_ = −0.274, *p* < 0.05). However, the social relationships domain did not show a significant correlation with absenteeism (r_(80)_ = −0.134, *p* > 0.05), indicating that this aspect of quality of life does not significantly impact absenteeism rates among caregivers.

## 4. Discussion

The findings of this study validate the intricate interplay between job satisfaction, quality of life (QoL), and absenteeism among formal caregivers, aligning closely with the hypothesized relationships outlined in the literature review. Consistent with previous studies, our results confirm that higher job satisfaction significantly correlates with better QoL across various domains, reflecting the crucial role of supportive workplace environments in enhancing caregiver well-being [19,20].

The positive correlations observed in Hypothesis 1 between job satisfaction and the QoL domains (physical, psychological, relationship, and environmental) support the argument that job satisfaction is a major contributor to the overall quality of life for caregivers [22]. The importance of this relationship between job satisfaction and quality of life underscores a clear connection between workplace experiences and the worker’s overall quality of life, influencing not only health and well-being, but also reducing employee turnover and absenteeism [15,17]. This result is particularly important as it highlights the need for healthcare policies aimed at improving job conditions for caregivers. Such policies would support the development of caregivers’ potential, ensure job suitability, and enhance professional satisfaction, thereby improving the quality of care provided to the elderly.

The support for Hypotheses 2a and 2b, which indicate that both overall and intrinsic job satisfaction are inversely related to absenteeism, aligns with previous research findings suggesting that higher job satisfaction can lead to reduced absenteeism [27,28]. However, the rejection of Hypothesis 2c highlights an important distinction: extrinsic factors, such as pay and workplace conditions, though influential, may not be sufficient on their own to reduce absenteeism without the support of intrinsic job satisfaction [36].

This suggests a complex dynamic in which internal factors of job satisfaction may play a more pivotal role in influencing absenteeism behaviors among caregivers. Absenteeism poses a critical challenge in fields that are inherently dependent on human intervention, such as healthcare caregiving. Our findings, therefore, underscore the importance for managers of healthcare units to prioritize factors that promote intrinsic satisfaction—such as professional achievement, autonomy and responsibility, recognition, and development opportunities—as a strategy to help reduce absenteeism rates.

Finally, the support for Hypothesis 3, with the exception of the social relationships domain, suggests that while certain aspects of quality of life help protect against absenteeism, social interactions among caregivers do not significantly impact absenteeism rates [19]. This exception may indicate that workplace social relationships function differently from other QoL domains, possibly due to unique stressors or the often-isolated nature of caregiving tasks.

Despite the valuable insights provided by the results, several limitations of this study must be considered. The sample size is a limitation, as it restricts the possibility of conducting more advanced statistical analyses and limits the generalizability of the findings. Additionally, the lack of access to qualitative data, such as participants’ personal narratives, may have reduced the depth of this study, as such data could have enriched the findings. Furthermore, the use of convenience sampling could introduce bias and diminish the generalizability of the results, as participants were selected from groups readily accessible to the researchers and may not accurately represent the broader population.

Future research could investigate specific aspects of social support that influence caregiver absenteeism, aiming to develop targeted interventions that address these unique challenges. Larger sample sizes in future studies would enhance external validity and allow for more robust statistical methods, such as regression analysis and Structural Equation Modeling (SEM). Additionally, longitudinal studies could offer deeper insights into how changes in job satisfaction over time impact both caregiver well-being and professional performance. Qualitative studies are also recommended, as they would give caregivers a voice, providing clearer insights into their perceptions and concerns.

The findings from this study have significant implications for managing caregiver resources, especially in the context of an aging global population. They suggest that interventions focused on enhancing job satisfaction—particularly through intrinsic rewards and improved training—could effectively reduce absenteeism and improve both caregivers’ quality of life and the quality of care provided to the elderly. From a policy perspective, these findings highlight the importance of healthcare policies that prioritize caregiver well-being through intrinsic rewards, structured support systems, and tailored training programs.

Policies that enhance intrinsic job satisfaction—through opportunities for professional growth, autonomy, and recognition—may help reduce absenteeism and improve both caregiver quality of life (QoL) and the quality of care provided to the elderly. Given the aging population and the increasing demand for elder care, investing in supportive policies could be essential for retaining skilled caregivers and ensuring sustainable, high-quality care.

## 5. Conclusions

In conclusion, this study enhances our understanding of the factors influencing the effectiveness and well-being of formal caregivers. Continued exploration of these relationships will enable stakeholders to better develop strategies that support the essential roles these professionals play in the lives of the elderly.

## Figures and Tables

**Table 1:** WHOQOL-Bref domains of quality of life: overall quality of life and general health (adapt from the WHOQOL Group, 1998) [35].

Physical Health	Pain and discomfort Sleep and rest Energy and fatigue Mobility Activities of daily living Dependence on medicinal substances and medical aids Work capacity
Psychological	Positive feelings Thinking, learning, memory and concentration Self-esteem Bodily image and appearance Negative feelings Spirituality/religion/personal beliefs
Social relationships	Personal relationships Social support Sexual activity
Environment	Freedom, physical safety and security Home environment Financial resources Health and social care: accessibility and quality Opportunities for acquiring new information and skills Participation in and opportunities for recreation/leisure activity Physical environment (pollution/noise/traffic/climate) Transport

**Table 2:** The characterization of the sample (N = 82), in percentage terms.

Gender	Male	6.1
	Female	93.9
	Not specified	-
Age	20–39	56
	40–59	25
	over 60	1
CMarital status	Single	37.8
	Married	47.6
	De facto marital status	8.5
	Separated	3.7
	Widowed	2.4
Ageing training	Yes	78.0
	No	22.0
Profession	Administrative	13.4
	Medical area	26.8
	Auxiliary areas	35.4
	Others	24.4

**Table 3:** Descriptive analysis of absenteeism variable (in days, N = 82).

	Minimum	Maximum	Mean	SD
Absenteeism	0	30	1.24	4.875

**Table 4:** Descriptive analysis of job satisfaction variable and its domains (N = 82).

	Mean	Median	Mode	SD
Intrinsic	3.85	3.90	3.90	0.73
Extrinsic	3.34	3.35	2.90	0.94
Overall	3.64	3.63	3.35	0.79

**Table 5:** Descriptive analysis of quality of life variable and its domains (N = 82).

	Mean	Median	Mode	SD
Physical	3.85	3.85	3.71	0.59
Psychological	3.82	3.83	3.67	0.65
Relationship	3.86	4.00	4.00	0.83
Environmental	3.66	3.69	3.50	0.61
Overall	3.80	3.79	4.01	0.55

**Table 6:** Pearson correlations between job satisfaction and quality of life and its domains (N = 82).

	Intrinsic	Extrinsic	Overall
Physical	0.383 ***	0.222 *	0.336 **
Psychological	0.389	0.274 **	0.370 **
Relationship	0.267 **	0.227 *	0.265 **
Environmental	0.508 ***	0.437 ***	0.515 ***
Overall	0.454 ***	0.343 ***	0.438 ***

* *p* < 0.05; ** *p* < 0.01; *** *p* < 0.001.

## Data Availability

The data used in the study are available on request at helena.martins@esce.ips.pt.
